# An actionable annotation scoring framework for gas chromatography-high-resolution mass spectrometry

**DOI:** 10.1093/exposome/osac007

**Published:** 2022-08-25

**Authors:** Jeremy P Koelmel, Hongyu Xie, Elliott J Price, Elizabeth Z Lin, Katherine E Manz, Paul Stelben, Matthew K Paige, Stefano Papazian, Joseph Okeme, Dean P Jones, Dinesh Barupal, John A Bowden, Pawel Rostkowski, Kurt D Pennell, Vladimir Nikiforov, Thanh Wang, Xin Hu, Yunjia Lai, Gary W Miller, Douglas I Walker, Jonathan W Martin, Krystal J Godri Pollitt

**Affiliations:** Department of Environmental Health Science, Yale School of Public Health, New Haven, CT, USA; Department of Environmental Science, Science for Life Laboratory, Stockholm University, Stockholm, Sweden; RECETOX, Faculty of Science, Masaryk University, Kotlarska 2, Brno, Czech Republic; Department of Environmental Health Science, Yale School of Public Health, New Haven, CT, USA; School of Engineering, Brown University, Providence, RI, USA; Department of Environmental Health Science, Yale School of Public Health, New Haven, CT, USA; Department of Environmental Health Science, Yale School of Public Health, New Haven, CT, USA; Department of Environmental Science, Science for Life Laboratory, Stockholm University, Stockholm, Sweden; National Facility for Exposomics, Metabolomics Platform, Science for Life Laboratory, Stockholm University, Solna 171 65, Sweden; Department of Environmental Health Science, Yale School of Public Health, New Haven, CT, USA; School of Medicine, Department of Medicine, Emory University, Atlanta, GA, USA; Icahn School of Medicine at Mount Sinai, Department of Environmental Medicine and Public Health, New York, NY, USA; Department of Physiological Sciences, Center for Environmental and Human Toxicology, University of Florida, Gainesville, FL, USA; Department of Chemistry, University of Florida, Gainesville, FL, USA; NILU—Norwegian Institute for Air Research, Kjeller , Norway; School of Engineering, Brown University, Providence, RI, USA; NILU—Norwegian Institute for Air Research, Framsenteret, Tromsø, Norway; MTM Research Centre, Örebro University, Örebro, Sweden; School of Medicine, Department of Medicine, Emory University, Atlanta, GA, USA; Mailman School of Public Health, Department of Environmental Health Sciences, Columbia University, New York, NY, USA; Mailman School of Public Health, Department of Environmental Health Sciences, Columbia University, New York, NY, USA; Icahn School of Medicine at Mount Sinai, Department of Environmental Medicine and Public Health, New York, NY, USA; Department of Environmental Science, Science for Life Laboratory, Stockholm University, Stockholm, Sweden; National Facility for Exposomics, Metabolomics Platform, Science for Life Laboratory, Stockholm University, Solna 171 65, Sweden; Department of Environmental Health Science, Yale School of Public Health, New Haven, CT, USA

**Keywords:** gas chromatography (GC), high-resolution mass spectrometry (HRMS), exposomics, chemicals, confidence scale, annotation

## Abstract

Omics-based technologies have enabled comprehensive characterization of our exposure to environmental chemicals (chemical exposome) as well as assessment of the corresponding biological responses at the molecular level (eg, metabolome, lipidome, proteome, and genome). By systematically measuring personal exposures and linking these stimuli to biological perturbations, researchers can determine specific chemical exposures of concern, identify mechanisms and biomarkers of toxicity, and design interventions to reduce exposures. However, further advancement of metabolomics and exposomics approaches is limited by a lack of standardization and approaches for assigning confidence to chemical annotations. While a wealth of chemical data is generated by gas chromatography high-resolution mass spectrometry (GC-HRMS), incorporating GC-HRMS data into an annotation framework and communicating confidence in these assignments is challenging. It is essential to be able to compare chemical data for exposomics studies across platforms to build upon prior knowledge and advance the technology. Here, we discuss the major pieces of evidence provided by common GC-HRMS workflows, including retention time and retention index, electron ionization, positive chemical ionization, electron capture negative ionization, and atmospheric pressure chemical ionization spectral matching, molecular ion, accurate mass, isotopic patterns, database occurrence, and occurrence in blanks. We then provide a qualitative framework for incorporating these various lines of evidence for communicating confidence in GC-HRMS data by adapting the Schymanski scoring schema developed for reporting confidence levels by liquid chromatography HRMS (LC-HRMS). Validation of our framework is presented using standards spiked in plasma, and confident annotations in outdoor and indoor air samples, showing a false-positive rate of 12% for suspect screening for chemical identifications assigned as Level 2 (when structurally similar isomers are not considered false positives). This framework is easily adaptable to various workflows and provides a concise means to communicate confidence in annotations. Further validation, refinements, and adoption of this framework will ideally lead to harmonization across the field, helping to improve the quality and interpretability of compound annotations obtained in GC-HRMS.

## Introduction

Environmental risk factors are major determinants of health, with current estimates suggesting that environmental exposures are responsible for about 23% of global disease burden,^1^ with chemical exposures responsible for one in six deaths.^2^ However, research has historically prioritized the characterization of genetic risk factors.^1^^,^^3-6^ Characterization of environmental factors primarily relies on self-reporting or community-level exposures.^3^ Identifying and preventing or reducing harmful environmental exposures could lead to significant reductions in global disease. Assessment of the chemical exposome, which includes the comprehensive identification of endogenous and exogenous compounds at the individual level, is essential to better understand the link between the environment and human health.^7^

Hundreds of thousands of chemicals are used in commercial and industrial applications. Once released into the environment, these chemicals often transform, increasing the number of potential chemical exposures to millions of compounds. Traditional methods for high-confidence annotation and quantitation require synthesized chemical standards for each analyte of interest, semi-manual peak integration, calibration curves, and often specific chromatographic methods. Therefore, based on cost and feasibility, the diversity of chemicals existing in the exposome presents a monumental challenge to traditional analytical methods, which typically interrogate less than 100 compounds.^8^^,^^9^ To overcome this limitation, non-targeted analytical methods that employ high-resolution mass spectrometry (HRMS) have been developed to screen and identify thousands of chemicals that may be present in environmental and biological samples. These methods use HRMS evidence indicative of universal chemical properties to annotate structure and estimate chemical abundances, and hence these methods are not solely focused on known or commonly expected chemicals. Because non-targeted results are more tentative than targeted results, a key need for such exposome research is to establish and apply transparency in instrumentation, data processing, and reporting criteria when communicating research findings.

Confirming the molecular structure of identified compounds using HRMS data remains a challenge. Due to limitations in scope and spectral quality of chemical databases and the difficulty of capturing mass fragmentation patterns for low abundance peaks, the confidence of chemical annotations can vary widely. Therefore, reporting may be limited to chemical formula, or even simply a set of retention time and mass spectral measurements, rather than an exact chemical structure. Chemical features that are not fully characterized can provide novel insights in exposomics research and links among environment, biological effects, and disease. For example, chemical formulas assigned to accurate mass features can aid in the characterization of unexpected and previously unknown chemical exposures that can be prioritized for study in other populations and sample types to understand their distribution in the environment. The advantage of using this approach for identifying environmental exposures is highlighted by the discovery of polychlorinated biphenyls (PCBs), which are one of the most widely studied persistent organic pollutants. Their initial discovery in environmental and biological samples was based upon detection of unknown peaks in multiple sample types, and it was their measurement across multiple samples and recognition of their accumulation in the food chain that led to the eventual identification of PCBs as highly persistent pollutants.^10^ More recent examples of the use of non-targeted mass spectrometry include the discovery of 6*p*-phenylenediamine(PPD)-quinone as a chemical responsible for fish die-offs,^11^ and products of a brominated pesticide transformation as responsible for eagle die-offs.^12^

When reporting annotations, it is essential to report measurements within a consistent framework that allows interpretation of the uncertainty in compound annotation. Defining a framework for reporting the confidence of chemical annotations is especially important given the potential for high false-positive rates and over-reporting in non-targeted and suspect screening HRMS studies across various classes of compounds.^13–16^ One of the first examples of a confidence scoring framework for non-targeted environmental chemical profiling was proposed by Schymanski *et al*.^18^ for liquid chromatography high-resolution tandem mass spectrometry (LC-HRMS/MS).^17^ Gas chromatography HRMS (GC-HRMS) provides complementary coverage of exposome chemicals that are of lower solubility, higher volatility, or that are not ionized by LC-HRMS approaches^19^ (Table S1). The applicability of Schymanski schema was reported to be limited for GC-HRMS due to differences in ionization, data acquisition, and data processing.^20^

Recently, a scoring framework for GC-HRMS using electron ionization (EI) was proposed^21^ categorizing confidence of feature identifications into four levels. Levels ranged from the most confident annotations in Level 1 with confirmed identifications to the weakest in Level 4 with features described only by exact mass. While this framework provides an important first step in defining scoring metrics for GC-HRMS, the parameters proposed for assigning confidence levels are potentially subject to high false-positive and false-negative rates. For example, Level-2 assignment requires isotopic distribution, while Level 3 is assigned for exact mass matches to fragment ions. The necessity of an isotopic pattern match requires the molecular ion, which is often not observed. Assignment of Level 3 is further challenged by the requirement of an isotopic pattern and exact mass matches that require exact mass libraries with known formulas, which are not currently available for many compounds. The schema does not incorporate retention index (RI), which we demonstrate to be an essential piece of information for reducing false positives. Thus, this approach may be suitable for databases with established, high mass-accuracy fragmentation spectra, but may be limited when applied to larger databases and in silico approaches.

Here, we present a new annotation confidence scoring framework specific for non-targeted GC-HRMS that can be used to determine and communicate confidence of chemical assignments made using several strategies. The framework draws on input from a community of GC-HRMS users and considers the breadth of chemical evidence that can be obtained from these powerful instruments. The current work focuses on environmental contaminants. While this framework can be applied to certain biological chemicals, the use of derivatization^22^ and class-based mass or fragmentation patterns may be required.

## Leveraging evidence from GC-HRMS to reduce false positives and negatives

GC-HRMS provides data-rich evidence that can be used to identify structural annotation of detected features, including various modes of ionization and fragmentation, accurate mass, retention information, and isotopic pattern. It is important to understand the unique advantages and limitations for each layer of evidence to decide the necessary pieces of information for accurate assignment of chemicals (Table S1).

### Ionization

The most common ionization method used in GC-HRMS is EI. Any molecule introduced to the source in this ionization mode is theoretically ionized with equal efficiency. In practice, however, on-column degradation, inlet/liner discrimination, and other factors^23^ can still cause total summed ion signal to be a factor of chemical structure.

EI is a “hard” ionization method, meaning that each molecule generates a spectrum with a high degree of fragmentation in which only traces of the intact molecular ion are usually detected, in most cases. In a GC–EI–MS analysis, each reported peak has an associated fragmentation spectrum, which is reconstructed by a deconvolution algorithm. Having fragmentation from all ions makes it possible to generate annotation levels for every sample, thereby improving confidence of chemical assignments. Generation of fragmentation spectra for all ionized compounds enables MS1 data to be used to confirm identified chemicals, eliminating the need for precursor selection and fragmentation by tandem mass spectrometry (MS/MS). To establish reproducible EI spectra libraries, ionization voltages of 70 eV are commonly used and combined with libraries containing RI. RI and fragmentation are often similar, irrespective of chromatographic method and instrument (within the same instrument type, eg, quadrupole time-of-flight MS [Q-TOF-MS]), and therefore have been successfully shared across laboratories.^24^

GC–EI–MS spectra provide key evidence for compound annotation, yet several challenges may still lead to false positives. While the diverse fragments observed in EI spectra allow for better structural characterization, the spread of signal across fragment ions can reduce overall sensitivity, often with no molecular ion signal present. Without observation of the molecular ion, many compounds can give similar spectra, even when they are not isomers. Because there is rarely a high signal from the molecular ion, ion selection followed by fragmentation cannot be applied in GC–EI–MS. Therefore, GC spectra must be deconvoluted across chromatographic time, often leading to the inclusion of artifact peaks due to high background or co-eluting compounds. Deconvolution can be a significant issue in increasing false negatives and false positives.

Alternative ionization strategies can be used with GC-HRMS to improve detection of the molecular ion, although these often lack the robustness, sensitivity, and reproducibility of EI. These techniques include positive chemical ionization (PCI), electron capture negative ionization (ECNI), and atmospheric pressure chemical ionization (APCI). The advantages and disadvantages of these techniques are described in the Supplementary Information.

### Accurate mass

Continual advances in high-resolution Orbitrap and Q-TOF-MS instrumentation have enabled accurate measurement of mass-to-charge ratios ranging from sub-ppm to 30 ppm mass accuracy windows, and resolutions up to 240,000, to date. Accurate mass (ability to measure the correct mass) and high resolution (ability to distinguish close masses) provide additional evidence for compound annotation, such as fine isotopic patterns. Unequivocal formula prediction may be possible for low-mass fragments, especially when including isotopic patterns. Isotopic pattern and mass defects can further be used to determine unique chemical structures containing certain functional groups (eg, Cl, Br, and F), aiding in the detection of unknown chemical exposures.

Suspect screening using accurate mass matching can reduce false positives. The largest GC–EI–MS libraries (eg, NIST and Wiley) include spectra acquired using unit mass instruments; only a handful of smaller libraries are available for spectral matching using accurate mass. Algorithms can be used to reduce false positives when matching experimental spectra obtained with GC-HRMS to unit resolution libraries.^25^ One example is applying a high-resolution mass filter (HRMF).^26^ The HRMF score corresponds to the percentage of fragment ions with formulae that can be predicted when setting atom constraints for formula-matching to include only those contained in the proposed molecular formula. Reverse HRMF (RHRMF) can also be used, but this approach limits scoring to only peaks found in the library, ignoring other peaks in the experimental spectra for scoring purposes. RHRMF reduces influences of artifacts during deconvolution on scoring, hence reducing false negatives, but may also increase false positives when experimental peaks are real and not found in the library.

### Retention index

Retention time is an orthogonal measurement that can improve annotation accuracy when combined with EI MS spectral matches. As retention time is influenced by column type, batch, and manufacturer as well as the method temperature gradient, standardized retention indices can be calculated using a series of compounds that span the entire chromatographic run (eg, alkanes,^27^ fatty acid methyl esters^28^). Retention indices can also be useful for identifying isomers with the same or similar EI spectra. Compared with LC, GC retention times are typically more consistent within and across laboratories.^29^^,^^30^ This increases the reliability of RI calibrations and enables matching of experimental retention indices to public libraries, as well as prediction of retention time indices using chemical properties.^31–33^

Retention indices provide key evidence to improve accuracy of compound annotation. There are a few considerations when implementing retention indices into a workflow. Methods using linear gradients may be preferable, to reduce errors in RI calculation,^34^ although non-linear temperature gradients may still be deployed with accurate RI calculated.^31^ While retention time indices improve annotation confidence, their incomplete availability in spectral libraries may limit their use. The largest libraries (eg, NIST and Wiley) have a high percentage of chemicals without RI information, limiting the search space and coverage if RI matching is required. The current release of NIST libraries contain predicted retention indices for many compounds; however, these predictions may not be accurate for all chemical classes.^32^ Careful consideration of retention time indices is also necessary depending on the chemicals being measured. For example, alkanes are not appropriate for calculating RI for persistent mobile organic compounds including per- and polyfluoroalkyl substances (PFASs), organophosphate esters (OPEs), and certain polar compounds amenable to GC.^35^^,^^36^

### Compound metadata for improving candidate ranking

While spectral and retention time information can be used to assign a structure or substructure to a chemical, additional evidence available from GC-HRMS can increase confidence of chemical identifications. Study context and likelihood of exposure are also important to consider. For example, a sizable portion of 100+ million chemical entries in PubChem have never been synthesized; their existence is limited to a patent or database,^37^ or if synthesized, were never generated at a large scale. Hence, tens of millions of these entries are irrelevant for exposomics studies and will lead to false-positive annotations.^38^ To reduce false positives and remove top-ranked compounds which are not likely to exist in the environment, more-selective databases, such as the EPA CompTox Chemistry Dashboard^39^ or PubChem Lite^38^ can be used. For GC-HRMS, a more actionable solution, given that the NIST and Wiley libraries are commonly used, is to include meta-data on database, patent and/or literature occurrence (reference counting), or production levels, for determining the likelihood that a chemical exists in the environment. This can drastically reduce false positives by removing unlikely candidates, and ease interpretation by retaining chemical species with known information.^39–41^ This method is biased to better-characterized chemicals, and any scoring methods are highly dependent on which databases or algorithms are used for determining compound occurrences. Hence, this method may lead to false negatives in the case of uncommon or unexpected chemicals, although the drastic reduction in false positives may far outweigh the potential concurrence of missed compounds.

There are several tools that can provide meta-data on a chemical’s likely occurrence, to improve non-targeted annotations in exposomics, including: NHANES Predicted Exposures,^41^ Data Sources,^40^ Number of PubMed articles containing chemical name, Number of PubChem data sources, and CPDat Product Occurrence Count.^40^

### Peak filtering to remove background contaminants and artifacts

Analytical artifacts can be introduced during sample collection, transport, storage, preparation, extraction, and acquisition, as well as through instrument noise. Complete physical removal of contaminants is not possible, particularly when background and artifact peaks overlap. Additional information, such as clustering of fragments across multiple samples, can be used to identify highly correlated fragments based on peak intensities. Matrix effects in GC^42^^,^^43^ are important for determining the extent of background noise. These matrix effects will depend on sample type, concentration, preparation, and introduction, as well as on the chromatography and acquisition strategies employed. These factors can impact spectral matches and annotation confidence and are challenging to incorporate into scoring metrics.

To account for these effects, blanks must be acquired and accounted for in any assignment of confidence. Various types of blanks can be used, including blanks collected in the field (field blanks), blanks carried out through all extraction, sample handling and storage (procedural blanks), solvent blanks, and instrument acquisition without injection. While the use of all these different blanks is important for diagnosing the source of background signal, for most purposes a comprehensive blank which covers all steps in which background can be introduced should be acquired. For example, stripped matrix or similar solvent should be introduced in collection vials during field work, and handled, shipped, and stored in the same manner as samples. These blanks should then be extracted using the exact same procedures during the same time(s) as the samples are being extracted. At least four blanks should then be stored identically and acquired on the instrument identically and be acquired throughout the acquisition to account for any changes in instrument background. After the proper blank is prepared and acquired, the data from the blank should be properly used to remove any background during data processing.

Multiple methods are available for blank filtering to remove background contaminants and artifacts.^44–49^ One approach is blank feature filtering where sample abundances are retained if above a blank threshold and has been found to outperform most other filtering methods.^45^^,^^48^ Blank feature filtering is ideally conducted using field blanks that have been treated identical to samples, undergoing the same transport, storage, sample preparation, and acquisition protocols. Using this method, the percentile or average of samples must be greater than the blank feature filter threshold (BFF_threshold_) calculated as

$${\text{BFF}}_{\text{threshold}}=c\;\times \left(B_{\text{average}}+3\times B_{\text{standard}\;\text{deviation}}\right),$$

where *c* is a constant (usually ranging between 2 and 10) and *B* is the blank signal.

A complimentary method to remove blank signatures is to look for signals that do not behave like well-retained compounds.^44^^,^^49^ Many background signals (eg, column bleed) will not form sharp peaks and/or will be highly variable in quality control samples injected across the instrument run. Consistency of deconvoluted spectra with respect to differing intensity in samples across batch in quality control samples or pools can therefore be used to remove background features (when many quality control samples are acquired). Furthermore, quality control calibration curves can be used to remove non-linear peaks, but this requires that the measured compounds are generally within the linear range in the quality controls.^47^ In this case, non-linear peaks at higher concentrations of the quality controls likely indicate that the source of the chemical is not from the sample, and if it is, that relative comparisons between samples are challenging. All these methods which do not implement a blank sample will not remove a well-behaved chromatographic peak which is a contaminant, and hence blank feature filtering is recommended as well, if these techniques are used.

Other methods can be used to generate a unique signature for compounds; therefore, rather than determine and remove blank signals, the analytes can be determined uniquely and retained. One such method is credentialing using isotopic labeling.^46^ This method incorporates isotopic labeling into cell cultures, and screens for the characteristic isotopic signals to only retain those metabolites which are generated in the experiment, effectively removing nearly all background. This method is specific to in vitro studies, and hence to only a subset of internal exposome studies.

## Compiling GC-HRMS evidence to communicate annotation confidence

There are three approaches for communicating confidence in annotations: non-probabilistic scores, probabilistic scores, and qualitative assignments of confidence. Individual layers of GC-HRMS evidence have community accepted standards for scoring. For example, EI match scores are generally calculated using dot-product or reverse dot-product, RI matches are usually based on an absolute deviation, meta-data scores are based on numbers of instances, and HRMF scores are based on a percentage of fragment formulae which can be predicted given the proposed structures composite atoms. Cut-offs for spectral match scores, RI delta, or other metrics based on experience, community accepted values, or optimized via experiments can then be assigned to remove compounds unlikely to be true positives.

**Figure 1.**
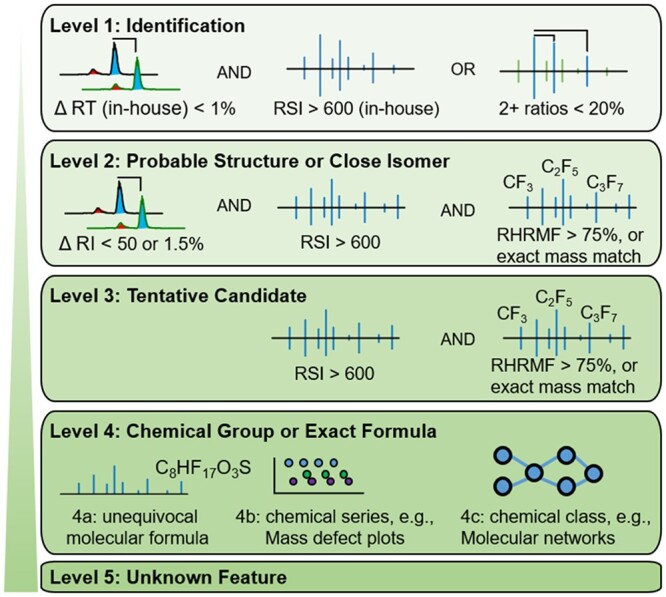
Proposed schema for assigning five levels of confidence in compound annotation using common evidence provided by GC-HRMS. Acronyms: retention time (RT), reverse search index (RSI), RI match (ΔRI), and RHRMF.

Combining different scores from various layers of GC-HRMS evidence to communicate confidence is non-trivial. Weighted linear models, neural networks, and mixed models, for example, have all been used to combine spectral similarity and RI information to optimize a single match function.^50^^,^^51^ Scores determined using these quantitative methods can improve candidate sorting compared with qualitative methods; however, statistical and coding experience are required which may be beyond the expertise of most users. Establishing harmonized algorithms and development of user-friendly software would ensure consistency across different user groups. Consistency across different software, workflows, and laboratories is much easier to achieve using qualitative assignment of confidence.^13^ Here, we introduce a qualitative means to assign and communicate confidence of feature annotations (Figure 1 and outlined below). We also show the use of more quantitative scoring metrics alongside qualitative methods for ranking candidates within a certain qualitative confidence assignment.

### Proposed system for assigning confidence

#### Level 1: Confirmed *identification* (retention time, EI spectra, and reference masses) using in-house library

- Retention time matches standard database generated in-house using the same method, background matrix, and instrument (spectra from certified reference standards, not from an external library). Retention times should match within the RSD of the associated standards (no more than 1% deviation).
- Observation of 2+ EI spectral reference peaks from standards at correct ratios (within 20%) or EI spectral match with in-house library >600. Often more than two reference masses and ratios are needed for confident annotation, 2 is minimum. For high-throughput screening with automated data extraction, verification of identity of each chemical in each sample by these criteria may be impractical. In such cases, procedures and assumptions should be clearly defined.

##### Advantage

- Confident identification for exact structure (with a few possible exceptions, especially enantiomers or structurally similar isomers which cannot be separated by retention time).

##### Limitation

- Expensive to purchase, prepare, run, and generate database for large number of chemicals. Standards may need to be reacquired each experiment if retention times are not stable between batches (eg, column clipping).
- Coverage is limited to standards available for purchase. Synthesized standards are often too costly for routine library building.

#### Level 2: Probable structure or close isomer using external libraries (RI match, [molecular ion], and EI match to exact mass library or including metrics incorporating accurate mass information)

- Reverse dot-product EI spectral match (>600) and dot-product (>500). As EI spectral prediction improves, predicted spectra may also be used.
- NCI/PCI/APCI when spectra contain more information than EI and at least five fragments are matched to a library database.
- RI match (<50 and <1.5%) or predicted RI (<100 difference) using validated methods.
- Match to an exact mass library (reverse dot-product >600) or using metrics incorporating exact mass (eg, high RHRMF score [>75]).

##### Advantage

- Correct annotation or the assigned structure is a structurally similar isomer (with certain exceptions).

##### Limitation

- RI match requirement may limit annotations if databases do not contain many compounds with RI. Therefore, this assignment of confidence is ideal when libraries have high RI coverage (experimental or predicted).
- In certain edge cases, RHRMF might lead to false positives when gas phase reactions in Orbitraps lead to fragments which cannot be predicted using molecular formula.

#### Level 3: Tentative candidate (EI accurate mass spectral match or EI match with metrics incorporating accurate mass) using external library; alternatively, RI match with accurate mass fragment matches

- Reverse dot-product EI spectral match (>600) and dot-product (>500). As EI spectral prediction improves, predicted spectra may also be used.
- NCI/PCI/APCI when spectra contain more information than EI and at least five fragments are matched to a library database.
- Match to an exact mass library (reverse dot-product >600) or using metrics incorporating exact mass (eg, high RHRMF score [>75]).
- Alternatively, when an RI match is within 100 (predicted or experimentally derived) but EI spectral match is based on accurate mass in silico spectra, rule-based presence or absence of ions (at least three, accurate mass), or observation of a molecular ion (accurate mass), a Level-3 assignment can be made.

##### Advantage

- Widest coverage while assigning a possible structure.

##### Limitation

- High rate of false positives (both in terms of exact structure and assigning a structurally similar isomer).
- Possibility of multiple peaks with similar fragmentation patterns (ie, structurally similar isomers), but different retention times.
- In certain edge cases, RHRMF might lead to false positives when gas phase reactions lead to fragments which cannot be predicted using molecular formula.

#### Level 4: Chemical group or exact chemical formula

##### Level 4A: Identification of unequivocal chemical formula from databases (PCI, NCI, or in certain cases, EI)

- Only one formula is possible given the exact mass of the precursor ion and isotopic distribution.
  - OR multiple formulas are possible, but after only including atoms which can predict all dominant fragment ions, only one formula is possible for the molecule.

##### Level 4B: Possible chemical series (eg, homologous series with repeating chemical constituents)

- Follows mass defect series (eg, using Kendrick mass defect^52^).
  - Homologous series have linear retention indices—so detection via GC can be precise—even for variable temperature programs.
- Has one or more fragments (with exact mass match) indicative of class.
  - OR shows defined subnetwork clustering in spectral similarity networks.

##### Level 4C: Possible chemical class (chemicals grouped based on structural motifs or similarity)

- Has one or more fragments (with exact mass match) indicative of class.
  - OR clusters in chemical similarity networks.
    OR other well-accepted class-specific method (eg, Lee index for polycyclic aromatic hydrocarbons [PAHs]).^53^^,^^54^

*Note:* Levels 4A–C are not necessarily in order of confidence or utility, for example in PFAS analysis the goal may be to find series of 3 or more compounds (Level 4B). In non-targeted analysis across all chemical classes molecular formula may be of more importance (Level 4A). Note that for Levels 4B and 4C, one could also have an exact formula match, these could be indicated as Level 4AB/4AC.

#### Level 5: Unknown feature (retention time and reference mass or deconvoluted spectra)

- Reproducibly detected deconvoluted spectra with reference mass intensity or combined intensity of all spectral ions. This feature may also have an annotation, and other information, but does not fit any of the criteria to be deemed Level 1, 2, 3, or 4.

### Important considerations

1. To avoid annotation of background artifacts, *peak filtering conditions must be met* for all confidence levels (eg, see discussion of blank feature filtering in the Supplementary Information).
2. If ion selection and fragmentation with CID/HCD is employed (eg, PCI, NCI, and APCI), then the Schymanski schema can be used to assign confidence levels.
3. For large studies, it is *important to account for spectral signals across multiple samples—*for example, was there only one good spectral match in one sample for a study with 300 samples? This case often indicates a false positive (larger sample size increases the number of detected features, enhancing the chance for matching noise in library spectra). However, it is critical to note that in exposomics studies, these features may represent unique exposures of interest. Multiple injections of a single sample, when possible, could aid in annotating rare chemical occurrences.
4. *In reporting results, it should be clear whether multiple structures have met the proposed criteria*. If more than one structure meets the criteria for an assignment of confidence level other information can be used to discern the likely correct annotation: for example, top hit has a significantly higher evidence count (by factor of 10), the compound is the only one expected for the matrix studied, the compound has a much more accurate RI (by 30), the compound has a significant higher reverse dot-product score (by at least 50), or the compound has a much more accurate RHRMF score (by at least 10). This is also where quantitative scoring metrics for ranking candidates can be utilized (total scores). Note that even if certain conditions above are met, there may still be false positives (see the “Results” section). If multiple top-hits still exist after applying our schema, the feature should be flagged accordingly.
5. While we have not made necessary observation of the molecular ion for Level 2, especially since there is a RI match, *observation of the molecular ion provides additional confidence* and should be achieved when possible.

### Comparison to the Schymanski schema for LC-HRMS/MS

While our schema (Figure 1) is based on the Schymanski schema for LC-HRMS/MS, it was modified to incorporate criteria specific to GC-HRMS. Included criteria were selected based on feasibility for replication across laboratories with the overall objective of implementing a harmonized scoring rubric. In addition, we include blank feature filtering, or similar, as a necessary criterion for all annotations, as well as chemical series and class-based annotation which is important in various fields.

Level 1—Criteria for GC-HRMS is similar to Schymanski Level 1 and provides compound identification. For GC-HRMS confidence scoring, the molecular ion and MS2 is not required due to fragmentation spectra available from MS1 when using EI.

Level 2—Criteria for GC-HRMS is similar to Schymanski Level 2; however, the use of retention indices has the potential to improve annotation confidence and assign unambiguous structures to isomers. The Schymanski schema states “spectrum–structure match is unambiguous” (Level 2A) or “represents the case where no other structure fits the experimental information” (Level 2B), which may be too stringent of an interpretation given the evidence provided. We would not confidently state this is true based on the suggested criteria for either LC or GC.

Level 3—Criteria for GC-HRMS fit within the Schymanski schema for Level 3, except predicted RI can be included as evidence for Level 2 given the accurate predictions.

Levels 4A–C—These levels represent unequivocal formulae and/or chemical series and chemical class, which are important in applications where the exact structure is not known but the series or formula provides valuable information. For example, these could be useful for polymers, PFAS, petrol chemicals, chlorinated by-products, and other applications where repeating series and discernable chemical classes of importance may be found. Unequivocal molecular formula is the same as Schymanski Level 4. It is useful to note that there was no place for series/determination of chemical class in the original Schymanski schema.

Level 5—This is different than in Schymanski Level 5 in that the exact mass of the feature cannot be discerned necessarily from EI spectra. Level 5 for GC-HRMS scoring to any reproducibly detected deconvoluted spectra which does not meet the criteria to be ranked in any other level and remains after filtering for artifacts and background contamination (eg, above blank signal thresholds).

## Methods: estimating false positives and false negatives for different confidence levels using an open-source software

We developed a software tool, Scoring, Integrating total spectral ion abundance, and Filtering for Gas Chromatography (SIF-GC), to automatically assign confidence levels to features identified in GC-HRMS data. The software performs all steps covered in the described scoring framework, providing the user with an output that includes RI, reverse similarity index, molecular ion, RHRMF (or similar), and integrated peak areas, heights, or total spectral ion abundance, for all samples and blanks. Our software initially assigns Level 2, 3 (not using the alternate evidence of RI and at least three accurate mass matches), or 5, and reports the top molecule in terms of a weighted score or other metric for each level. The software also indicates whether multiple hits exist for each level (Level 1 is reserved for manual targeted techniques and Level 4 may be project-specific and is not implemented at this time). The software then can be used to apply blank feature filtering (user modifiable parameters), and further filter features to only retain compounds with unique CAS-RN or other identifiers and compound names (removing duplicates). Furthermore, the SIF-GC software can be used to compute total spectral ion abundance for all compounds given an average ratio of total spectral ion abundance to peak area or height. All steps are automated for Thermo Compound Discoverer outputs, but they could also work, with some minor adjustments, with other vendor platforms. The SIF-GC software is open source and freely available at innovativeomics.com/software/.

To evaluate the performance of our SIF-GC software, and our schema for reporting confidence, we applied the tool to datasets acquired from biological and environmental samples and investigated the false-positive and false-negative rates assigned for each level. The first dataset was of human serum samples spiked with 112 standards (90 native and 22 isotopic labeled chemicals; Table S1A), with a final concentration range of 6.7–33.6 ppb. The spiked serum validation dataset consisted mainly of brominated flame retardants, chlorinated flame retardants, organochlorine pesticides, PAHs, oxygenated PAHs, nitrated PAHs, PCBs, polychlorinated dibenzodioxins, and polychlorinated dibenzofurans. The second dataset describes indoor and outdoor air samples collected using passive samplers from homes near natural gas compressor stations. A targeted panel of 81 standards (70 native and 11 isotopic labeled chemicals; Table S1B) with a final concentration range of 32–1000 ppb was assessed for these environmental samples. This validation set consisted of alkaloid, benzodioxoles, benzopyrans, brominated flame retardants, chlorinated hydrocarbons, haloethers, nitroaromatics, nitrosamines, phthalates, pyrethroids, organochlorine pesticides, organophosphates, PAHs, PCBs, and volatile organic compounds. We compared compounds assigned Level 1 (targeted methods used to confidently assign compounds) with compounds assigned Level 2, 3, or 5 using the SIF-GC software. A list of experimental protocols is provided in the Supplementary Information.

## Results: how confident are the confidence levels?

**Figure 2.**
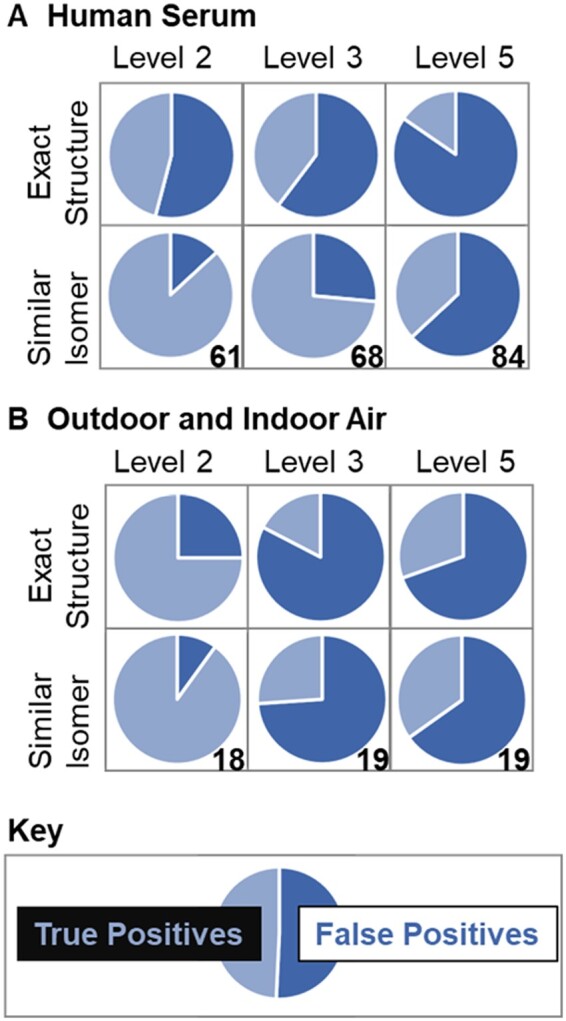
False-positive rates and number of assignments across each confidence level for 81 observed unlabeled standards spiked into human serum (**A**), and 19 Level-1 assignments from outdoor and indoor air samples (**B**).

We selected qualitative criteria that can be readily implemented given common metrics provided by most GC open source and vendor library search software. These common metrics included RI (delta), spectral match (in this case, reverse similarity index was used), and a metric incorporating exact mass (in this case, RHRMF criteria was used). Using the SIF-GC software, we determined the false negative, false positive, and true positive rate when utilizing our criteria for assigning confidence at Levels 2 and 3. For the *human serum samples*, of the 90 spiked unlabeled standards which were detected by GC-HRMS, 80 were deconvoluted by Thermo Compound Discoverer. This highlights a potential source of false negatives; molecules of interested might not have associated deconvoluted spectra when ion abundances are low, peak shape is poor or there is spectral overlap from other ions, background interferences are high, or other thresholds specified by deconvolution algorithms are not met. Of these 80 chemicals, 61 were assigned a Level-2 annotation (76%), and of these Level-2 annotations, the correct exact structure was only identified for 28 compounds (46%). Hence, in absolute terms, using our scoring criteria, the highest level of confidence for suspect screening, there was a 54% false positive for Level-2 assignments, 60% false-positive rate for Level-3 assignments, and 84% false-positive rate for top-ranked assignments without Level-2 or -3 criteria (Figure 2A). Of the deconvoluted spiked standards, 19 (24%) had no Level-2 assignment and hence were considered false negatives. In many cases, these false negatives are due to unavailable libraries or RI values for the standard.

For *air samples*, there was a 22% false-positive rate for Level-2 assignments, 89% false-positive rate for Level-3 assignments, and 74% false-positive rate for top-ranked assignments without Level-2 or -3 criteria (Figure 2B). It should be noted there was a lower false-positive rate in air samples compared with the human serum samples. This difference is attributable the standards used with each sample type: serum samples were spiked with a mixture that was mainly comprised of halogenated isomers which could not be distinguished by RI and EI spectra, whereas the air samples included a wider range of chemicals. Therefore, for exact structure, the air samples may be more representative of the false-positive rate for Level 2 when the range of chemicals being annotated is a cross a diverse array of structures, many of which do not have close isomers.

**Figure 3.**
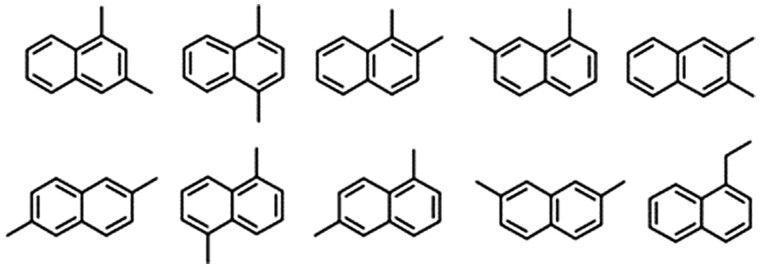
Examples of 10 isomers (dimethyl naphthalene and ethyl naphthalene) which were all assigned Level 2 for a single feature. The dimethyl naphthalene species represented one of the highest abundance features in air samples.

Even when using RI, RHRMF, reverse search index, molecular ion, and search index there are subtle isomeric differences (eg, Figure 3), the exact structure often cannot be discerned using GC–EI-HRMS (hence, the 54% false-positive rate in serum samples and 22% false-positive rate in air samples). When subtle isomeric differences in the position of methyl, chlorine, heteroatoms, and aromatic rings were not considered false positives, 13% of the 61 top-ranked Level-2 assignments in the *spiked serum* were false positives, 25% of the top-ranked Level-3 assignments were false positives, and 61% of the top-ranked assignments without Level-2 or -3 criteria (Level 5) were false positives (Figure 2A). Similarly, when not considering highly similar isomers false positives, for *air samples*, 11% of the 18 top-ranked Level-2 assignments were false positives, 79% of the top-ranked Level-3 assignments were false positives, and 68% of the top-ranked assignments without Level-2 or -3 criteria (Level 5) were false positives (Figure 2B).

Application of the proposed GC-HRMS scoring framework in datasets from biological and environmental samples highlighted that: (1) RI is essential for confident assignments and (2) structurally similar isomers often cannot be resolved even with a RI match. Exact mass filters (eg, RHRMF used here) were also shown to be valuable, especially when heteroatoms exist. There were 26% false positives using RHRMF (Level 3) compared with the 63% false-positive rate identified without any filters for the validation set containing mostly chlorinated species (Figure 2A). RHRMF was also found to be valuable in the validation set containing mostly phthalates and PAHs (Table 1).

**Table 1.** Summary of confidence in annotated chemicals detected in outdoor and indoor air samples

Unlabeled standard	Number of candidates							Correct Level-2 Assignment	Molecular ion observed for correct candidate
	Total detected	Level 3, all criteria	Level 2, all criteria	Level 2, RI criteria	Level 2, reverse search index criteria	Level 2, RHRMF criteria	Correct molecular ion observed		
Benz[a]anthracene	25	14	3	5	18	16	11	Yes	Yes
Chrysene	39	19	3	4	33	24	11	Yes	Yes
Fluoranthene	54	28	9	12	34	43	6	Yes	Yes
Fluorene	33	29	7	9	32	29	7	Yes	Yes
Acenaphthylene	33	23	7	12	33	23	5	Yes	Yes
Naphthalene	40	32	8	11	40	32	9	Yes	Yes
Phenanthrene	38	38	7	7	38	38	13	Yes	Yes
Pyrene	53	23	7	7	36	34	6	Yes	Yes
2-Chloronaphthalene	27	13	2	6	27	13	3	Yes	Yes
Dibenzofuran	49	29	5	16	48	29	12	Yes	Yes
Hexachlorobenzene	32	9	6	12	31	9	5	Yes	Yes
Hexachlorobutadiene	32	2	1	2	26	2	1	Yes	Yes
Tris(1-chloro-2-propyl) phosphate	20	5	3	7	17	7	0	No	No
4,4′-Dibromoocta-fluorobiphenyl	27	1	1	2	27	1	1	Yes	Yes
Isophorone	49	46	19	19	49	46	8	Yes	Yes
*N*-Nitroso-diphenylamine	46	35	4	4	46	35	23	No	No
Butylbenzyl phthalate	21	7	4	7	18	7	3	Yes	No
Diethyl phthalate	28	18	4	6	27	18	0	Yes	No
Di-*n*-octyl phthalate	53	45	9	10	52	45	1	No	No
Percent filtered		44 ± 28	84 ± 8	76 ± 10	9 ± 12	40 ± 27	83 ± 14		

Individual layers of GC-HRMS evidence were not sufficient at differentiating between similar chemicals. Detected chemicals were filtered based on multiple criteria including RI, reverse search index, RHRMF, and observation of the molecular ion. The number of retained candidates after applying all criteria for Levels 2 and 3 is shown for individual chemicals as well as each Level-2 criteria. The overall percent of candidates removed is also presented.

A summary of each annotation filter, the number of candidates retained, and the percentage of candidates removed after applying the filters for air samples is shown in Table 1. The individual filters which removed the most candidates on average were requiring molecular ion (83% ± 14% removed), followed by RI match < 100 (76% ± 10% removed), RHRMF > 75% (40% ± 27% removed), and reverse search index (40% ± 12% removed) (Table 1). These findings highlight the importance of the RI criteria for removing false positives. When RI is combined with RHRMF and reverse search index for a Level-2 assignment, 84% ± 8% candidates were removed on average. Hence, combining these three filters further lowers the false-positive rate. While use of molecular ion was the most stringent filtering criterion, reducing false positives, decreasing the false-positive rate must be balanced against the increase in false negatives (number of correct candidates removed using the respective filter). Use of the molecular ion drastically increased false negatives.

False negatives were designated when the correct assignment (exact structural match) was removed by the respective filter. Requiring the molecular ion led to 5 false negatives out of the 19 validated assignments (26%; Table 1), use of RI led to 11% false negatives (2/19), reverse search index to 5% false negatives (1/19), and RHRMF to 0% false negatives (data not shown). When RI, reverse search index, and RHRMF were combined (Level-2 assignment), there were 16% false negatives (3/19; Table 1). The false negative rate for requiring molecular ion may be even higher, as this validation set consisted of PAHs and derivatives (over 50% of validation set); PAHs have a high molecular ion signal. Therefore, the generalized criteria we present do not necessitate the molecular ion (RI removes many of the same candidates and the false-negative rate of requiring molecular ion is too high), but depending on chemical class, for example when looking at PAHs, necessitating molecular ion may be a beneficial additional filter. When adding molecular ion as a requirement for Level-2 assignments, a slight decrease in false positives was observed, but a significant increase in false negatives was also observed (Figure S1).

## Concluding remarks: utility, potential challenges, and future work

It is important to note that the assignment of confidence is based on filters, and that often, multiple candidates remain even after stringent filtering criteria. For example, in the outdoor and indoor air dataset, only 2 of the 19 features with a Level-1 assignment had a single candidate retained after Level-2 filtering, while the remaining 89% of assigned features had more than one Level-2 candidate (Table 1). Current filter cutoffs are voluntaristic, based on expert consensus. Optimization of filters may further reduce false positives, while limiting false negatives, but this will be compound-class and workflow specific. Furthermore, multiple candidates will always exist for multiple features, regardless of thresholds used to keep false negatives low. Therefore, ranking algorithms are essential. When adding the Level-2 criteria to the ranking algorithm from Thermo Compound Discoverer, a significant number of false positives were removed without a significant increase in false negatives, for both validation datasets (Figure 2). Using search index scores for ranking (eg, dot product) performed similarly, and hence simple scoring metrics can be chosen which are not vendor-specific. Further ranking based on meta-data can also be beneficial using information such as database occurrence. For example, in the air samples evaluated, N,N-diethyl-meta-toluamide (DEET) was observed, but a close isomer which is not commonly found in the environment was ranked first, while DEET was ranked second (both Level-2 assignments). Database matching could be used to rank DEET higher; this compound was expected, as individuals residing in residences where air samples were collected reported use of this insect repellant.

We outline a qualitative scoring framework for assigning confidence to chemical assignments in GC-HRMS data. The proposed schema was developed to be actionable with easy implementation using most deconvolution and annotation algorithm outputs. Furthermore, we introduce an open access software tool (SIF-GC) to automate feature scoring. The assignment of confidence follows a five-level scoring framework that has been well-established by Schymanski et al. for LC-HRMS/MS. The criteria used to determine confidence for GC-HRMS are common metrics in the GC field, including blank filtering, (reverse) spectral matches, (R)HRMFs or exact mass spectral matches, RI, and molecular ion. Application of the proposed scoring system to environmental and biological datasets resulted in a false-positive rate of 11% and 12%, respectively, for Level-2 assignments, the highest level which can be assigned when using suspect screening approaches based on our schema. The false-negative rate (removal of the correct candidate) for Level-2 assignments was found to be 16% for the air samples and 24% for the spiked human serum. When close isomers (eg, alternate positions of chlorine atom(s) on PCBs, or methyl groups on PAHs) are considered false positives, this false-positive rate becomes much higher (22% for air samples and 54% for human serum). Hence, when using suspect screening for GC, RI alone could not distinguish close isomers in most cases. In this case, elution order, rather than RI, can be valuable, although this is difficult to automate and implement on a large scale.

While Level-2 assignments can provide relatively confident annotations, at least at the level of isomers with subtle difference from the exact structure, Level-3 assignments were more tentative, and confidence varied widely depending on structure. Nonetheless, Level-3 criteria improved assignment confidence over not using any criteria when chemicals with heteroatoms are investigated. Furthermore, Level-4 assignments can be used to assign chemical classes, when for example, all PAHs are of interest, even when exact structure cannot be determined. It is important to note that depending on matrix, acquisition methods, and analyte classes, different false-positive and false-negative rates may be determined. Future work applying the confidence levels presented here to standard reference material could be used to define quantitative levels of confidence a priori, eg, Level 1—95%, Level 2—80%, and Level 3—50%.

The use of filtering criteria decreased the false-positive rate when compared with common ranking algorithms alone (eg, Thermo Compound Discover’s ranking algorithm). For the >100 molecules validated in this study, use of RI, reverse search index, and reverse high-resolution mass filtering did not substantially increase false negatives. Therefore, Level-2 annotations can be exclusively considered for many suspect screening applications. The requirement of molecular ion for EI increased false negatives significantly and is only recommended for certain chemical classes that are highly stable (eg, most PAHs). It is important to mention that the use of accurate mass—either through molecular ion accurate mass matches, high-resolution mass spectral libraries, RHRMFs, or other techniques—is beneficial for reducing false positives. We did not discern between instruments with different resolving power (our validation datasets were acquired at 40 000 and 60 000 for the air and serum samples, respectively), but this may influence the false-positive and false-negative rates when using Level-2 and Level-3 filters. Furthermore, Orbitraps and other trap instruments may generate ions that cannot be predicted from molecular formula, and hence Q-TOFs may have a lower false-negative rate in this regard.

While the criteria included in the proposed GC-HRMS scoring framework substantially decreased false positives and can be used to assign more confident annotations, other metrics may also be explored, including chemical database occurrence (higher occurrence means it may be more likely to exist in samples), peak shape, and use of ECNI, PCI, APCI, and other ionization techniques for validation. Database matching may be especially helpful when close isomers are observed, but only a common isomer exists. Furthermore, even after Level-2 filtering, multiple candidates were retained in nearly all cases. Therefore, more quantitative scoring metrics for ranking need still be applied to determine the correct candidate. We find that the total score provided by Compound Discoverer, or simply using reverse search index to rank candidates, works well in tandem with Level-2 filters. The Level-2 filters were further found to reduce false positives substantially when using either of these ranking algorithms, than when using that ranking algorithm alone. Furthermore, it is important to note that the cutoffs for scoring metrics (RI and EI spectral match) presented herein may be refined by future studies. For example, studies which comprehensively examine all thresholds for a broad range of spectral data to determine optimal cutoffs that would reduce false positives and false negatives could be incorporated. Other criteria that may help to increase identification confidence is the addition of context-dependent metadata (eg, sample source) and isotopic ratios. We encourage any future addendum to this article that suggests other optimal cutoffs and includes additional criteria for identification.

The proposed confidence scores provide a strategy for communicating levels of evidence that were used to predict annotation of chemicals detected using non-targeted analysis. While false positives were still present across all annotation levels, this approach assists in identifying the likelihood that a match is correct and can be used to facilitate selection of potential compounds for further evaluation and validation using additional analytical techniques. Level-1 assignment (using standards) is still necessary in most work with direct regulatory and policy implications. This scoring framework can be easily extended to GC-HRMS data for exposome analysis and provides a foundation for linking annotations performed across multiple laboratories and studies, and for building cumulative databases.

The framework for confidence reporting is essential for the field to progress from fragmented study-specific outputs toward comparative reporting that can be harmonized across studies. This is only one aspect of workflow harmonization in non-targeted analysis. Where possible, community accepted guidelines or best practices, and robust quality control procedures across the entire non-targeted workflow are needed. Multiple institutions and organizations are working toward this end including The European Partnership for the Assessment of Risks from Chemicals, Environmental Exposure Assessment Research Infrastructure, Benchmarking and Publications for Non-Targeted Analysis, and Metabolomics Quality Assurance and Quality Control Consortium. These procedures will enhance interpretation and cross-laboratory integration of data, improve data quality (eg, less false positives and negatives) and transparency, and allow for more rapid adoption in clinical, regulatory, and other applied frameworks.

## Supplementary Material

osac007_Supplementary_Data

## Data Availability

Annotated results and vendor raw files can be downloaded at the following link: ftp://massive.ucsd.edu/MSV000090537/ (note certain browsers including microsoft edge will not work, it is recommended to use ftp or Firefox web browser).
