# Supplementary material for: An actionable annotation scoring framework for gas chromatography-high-resolution mass spectrometry
Source: Exposome. 2022 Aug 25;2(1):osac007. doi: 10.1093/exposome/osac007 (PMC9719826; doi:10.1093/exposome/osac007)
Supplement: osac007_Supplementary_Data [file osac007_supplementary_data.docx]

**Supplemental Information**

**An Actionable Annotation Scoring Framework for Gas Chromatography - High Resolution Mass Spectrometry (GC-HRMS)**

Jeremy P. Koelmel^1^, Hongyu Xie ^2^, Elliott J. Price^3^, Elizabeth Z. Lin^1^, Katherine E. Manz^4^, Paul Stelben^1^, Mathew K. Paige^1^, Stefano Papazian^2^, Joseph Okeme^1^, Dean P. Jones^5^, Dinesh Barupal^5^, John Bowden^11,12^, Pawel Rostkowski^6^, Kurt D. Pennell^4^, Vladimir Nikiforov^7^, Thanh Wang^8^, Xin Hu^10^, Yunjia Lai^9^, Gary W. Miller^9^, Douglas I. Walker^5*^, Jonathan W. Martin^2^, and Krystal J. Godri Pollitt^1*^

^1^ Department of Environmental Health Science,
Yale School of Public Health, New Haven, CT, 06520, United States

^2^ Department of Environmental Science, Science for Life Laboratory,
Stockholm University, Stockholm 10691, Sweden

^3^ RECETOX, Faculty of Science, Masaryk University, Kotlarska 2, Brno, Czech Republic

^4^ School of Engineering, Brown University, Providence, RI 02912, United States

^5^ Department of Medicine, School of Medicine,
Emory University, Atlanta 30322, United States

^6^ Department of Environmental Medicine and Public Health,
Icahn School of Medicine at Mount Sinai, New York, NY, 10029, United States

^7^ NILU- Norwegian Institute for Air Research, 2007 Kjeller, Norway

^8^ NILU- Norwegian Institute for Air Research, Framsenteret, 9007 Tromsø, Norway

^9^ MTM Research Centre, Örebro University, SE-701 82, Örebro, Sweden

^10^ Department of Environmental Health Sciences, Mailman School of Public Health, Columbia University, New York, NY 10032, United States

^11^ Center for Environmental and Human Toxicology & Department of Physiological Sciences, University of Florida, Gainesville, FL, 32603, United States

^12^ Department of Chemistry, University of Florida, Gainesville, FL, 32603 United States

*Corresponding authors

**Krystal J. Godri Pollitt**

Laboratory of Epidemiology and Public Health,

60 College Street, Room 523, New Haven, CT, 06520

[krystal.pollitt@yale.edu](mailto:krystal.pollitt@yale.edu)

**Douglas I. Walker**

Atran Berg Laboratory Building,

1428 Madison Ave, Floor 3 Room 39, New York, NY, 10029

[douglas.walker@mssm.edu](mailto:douglas.walker@mssm.edu)

**S1. Leveraging Evidence from GC-HRMS to Reduce False Positives and Negatives**

**Alternative EI spectral libraries and ionization methods to improve coverage**

For the discovery of unknown unknowns where no libraries or standards exist, the in-silico prediction of EI spectra (Allen et al., 2016; Spackman et al., 2018; Wang et al., 2020; Wei et al., 2019) is more straight forward than collision-induced dissociation (CID) (Koopman and Grimme, 2021) and higher energy C-trap dissociation (HCD) spectra used for LC-HRMS analysis. Although, it is important to recognize that false positive rates vary across all in silico approaches (Schreckenbach et al., 2021). In addition to predicted spectral matching, EI spectra can be used for molecular networks and substructure characterization (e.g., (Elie et al., 2019; Hummel et al., 2010; Lai et al., 2018; Stein, 1995)). The high degree of information contained in EI fragmentation reduces the number of similar spectral matches and allows for detailed substructural analysis (e.g., chemical similarity networks).

Alternative ionization strategies can be used with GC-HRMS to improve detection of the molecular ion, although these often lack the robustness, sensitivity, and reproducibility of EI. Additionally, for high-throughput screening the use of alternative ionization techniques *in addition to EI* may be cost prohibitive. These techniques include positive chemical ionization (PCI), electron capture negative ionization (ECNI), and atmospheric pressure chemical ionization (APCI). Unlike EI, the ionization efficiency for PCI, ECNI, and APCI is dependent on chemical structure. While ECNI is the least universal ionization technique (in terms of ionization efficiencies across chemical classes), this approach can be highly selective and provide high sensitivity for ions which are formed favorably by electron capture (e.g., halide containing compounds such as chlorinated and brominated byproducts). PCI can be adapted for various chemical analytes because reagent gases are ionized and used to transfer positive charge to the analytes. Whereas methane gas is usually used, other reagent gases can be selected to increase sensitivity for specific classes of molecules (Little and Howard, 2013). APCI can provide higher sensitivity for certain molecules, for example, negative mode APCI, like ECNI, can reveal halogenated compounds (such as halogenated paraffins) that standard GC-EI-MS misses. While these methods can be challenging to implement in standardized nontargeted workflows, they provide an important alternative ionization method for detection molecular ions and predicting potential formulas in the identification of unknowns.

**S2. Methods: Acquisition of gc-hrms datasets**

**S2.1. Acquisition of the human serum validation dataset**

The GC-HRMS dataset of human serum samples was obtained by spiking 112 standards (90 native and 22 labeled chemicals) to commercial pooled human serum (Type AB, Sigma-Aldrich, Germany), and 500 µL aliquots were extracted after removal of proteins and lipids at Stockholm University. After spiking with the standard mixture (list in **Table S1A**), the extracts were analyzed by GC-HRMS (Q Exactive GC Orbitrap, Thermo Scientific) using electron ionization (EI) in full scan mode (44–750 *m/z*, 60,000 resolution FWHM at 200 *m/z*). The injection volume was 1 µL for each run. A capillary DB5 column (30 m × 0.25 mm × 0.25 µm film thickness) was used with the temperature program: 50 °C held for 1 min, ramped 10 °C min^−1^ to 315 °C and held for 8 min.

**S2.2. Acquisition of the outdoor and indoor air validation dataset**

Passive air samplers (Fresh Air Clips) were deployed inside and outside homes proximal to a natural gas compressor station in Jefferson County, Ohio. Methods have been previously described (Martin et al., 2021). Briefly, airborne containments were collected using polydimethylsiloxane (PDMS) sorbent bars that were housed in a polytetrafluoroethylene (PTFE) chamber. This assembly was mounted in a magnetic clip under a weather cover during the sampling period. PDMS sorbent bars were cleaned at the Yale School of Public Health and shipped together with other materials to the study site. Four cleaned PDMS sorbent bars were placed into the PTFE chamber and deployed at indoor and outdoor locations at the home of study participants. At the end of the sampling period, passive air samples were collected, shipped back to the Yale School of Public Health using cold chain transport, and stored at -80 °C until analysis. Samples were spiked with a mixture of labelled standards and extracted by thermal desorption (Gerstel TDU) and analyzed by GC-HRMS (Q Exactive GC Orbitrap, Thermo Scientific) using electron ionization (EI) in full scan mode (53–800 *m/z*, 40,000 resolution FWHM at 200 *m/z*). A capillary TG-5SILMS column (30 m × 0.25 mm × 0.25 µm film thickness) was used with the temperature program: 70 °C held for 1 min, ramped 7 °C min^−1^ to 300 °C and held for 4 min. A total of 81 chemicals were assessed (**Table S1B**).

**Table S1A. List of native and isotopic labeled standards spiked into human serum samples.** Acronyms: brominated flame retardants (BFR); chlorinated flame retardants (CFR); organochlorine pesticides (OCP); polycyclic aromatic hydrocarbons (PAH); oxygenated polycyclic aromatic hydrocarbons (oxy-PAH); nitrated polycyclic aromatic hydrocarbons (nitro-PAH); polychlorinated biphenyls (PCB); polychlorinated dibenzodioxins (PcDD); polychlorinated dibenzofurans (PcDF).

| **Chemical Class** | **Detected Metabolite** | **CAS-RN** | **Molecular Formula** | **Concentration (ppb)** |
| --- | --- | --- | --- | --- |
| BFR | 2,4,4´-Tribromodiphenyl ether (BDE-28) | 41318-75-6 | C12H7Br3O | 16 |
| BFR | 2,2´,4,4´-Tetrabromodiphenyl ether (BDE-47) | 5436-43-1 | C12H6Br4O | 16 |
| BFR | 2,3´,4´,4-Tetrabromodiphenyl ether (BDE-66) | 189084-61-5 | C12H6Br4O | 16 |
| BFR | 2,2´,3,4,4´-Pentabromodiphenyl ether (BDE-85) | 182346-21-0 | C12H5Br5O | 16 |
| BFR | 2,2´,4,4´,5-Pentabromodiphenyl ether (BDE-99) | 60348-60-9 | C12H5Br5O | 16 |
| BFR | 2,2´,4,4´,6-Pentabromodiphenyl ether (BDE-100) | 189084-64-8 | C12H5Br5O | 16 |
| BFR | 2,2´,4,4´,5,5´-Hexabromodiphenyl ether (BDE-153) | 68631-49-2 | C12H4Br6O | 16 |
| BFR | 2,2´,4,4´,5,6´-Hexabromodiphenyl ether (BDE-154) | 207122-15-4 | C12H4Br6O | 16 |
| CFR | Dechlorane 603 | 13560-92-4 | C17H8Cl12 | 16 |
| OCP | Pentachlorobenzene (PeCB) | 608-93-5 | C6HCl5 | 16 |
| OCP | alpha-Hexachlorocyclohexane (α-HCH) | 319-84-6 | C6H6Cl6 | 16 |
| OCP | Hexachlorobenzene (HCB) | 118-74-1 | C6Cl6 | 16 |
| OCP | beta-Hexachlorocyclohexane (β-HCH) | 319-85-7 | C6H6Cl6 | 16 |
| OCP | gamma-Hexachlorocyclohexane (γ-HCH) | 58-89-9 | C6H6Cl6 | 16 |
| OCP | delta-Hexachlorocyclohexane (δ-HCH) | 608-73-1 | C6H6Cl6 | 16 |
| OCP | Heptachlor | 76-44-8 | C10H5Cl7 | 16 |
| OCP | Aldrin | 309-00-2 | C12H8Cl6 | 16 |
| OCP | Heptachlor epoxide B | 1024-57-3 | C10H5Cl7O | 16 |
| OCP | Heptachlor Epoxide A | 28044-83-9 | C10H5Cl7O | 16 |
| OCP | β-chlordane(trans-Chlordane) | 5103-74-2 | C10H6Cl8 | 16 |
| OCP | O, P’-DDE | 3424-82-6 | C14H8Cl4 | 16 |
| OCP | α-chlordane(cis-Chlordane) | 5103-71-9 | C10H6Cl8 | 16 |
| OCP | trans-Nonachlor | 39765-80-5 | C10H5Cl9 | 16 |
| OCP | p,p’-DDE | 72-55-9 | C14H8Cl4 | 16 |
| OCP | Dieldrin | 60-57-1 | C12H8OCl6 | 16 |
| OCP | O, P’-DDD | 53-19-0 | C14H10Cl4 | 16 |
| OCP | Endrin | 72-20-8 | C12H8Ocl6 | 16 |
| OCP | p,p’-DDD | 72-54-8 | C14H10Cl4 | 16 |
| OCP | cis-Nonachlor | 5103-73-1 | C10H5Cl9 | 16 |
| OCP | o,p’-DDT | 789-02-6 | C14H9Cl5 | 16 |
| OCP | p,p’-DDT | 50-29-3 | C14H9Cl5 | 16 |
| Oxy-PAH | Phenalen-1-one | 548-39-0 | C13H8O | 16 |
| Oxy-PAH | Anthracene-9,10-dione | 483-35-2 | C14H8O2 | 16 |
| Oxy-PAH | 4H-Cyclopenta[def]phenanthrene-4-one | 5737-13-3 | C15H8O | 16 |
| Oxy-PAH | 2-Methylanthracene-9,10-dione | 84-54-8 | C15H10O2 | 16 |
| Oxy-PAH | 9H-Fluoren-9-one | 486-25-9 | C13H8O | 16 |
| Nitro-PAH | 1-Nitro naphthalene | 86-57-7 | C10H7NO2 | 18 |
| PCB | 2-Chlorobiphenyl (PCB 1) | 2051-60-7 | C12H9Cl | 16 |
| PCB | 4-Chlorobiphenyl (PCB 3) | 2051-62-9 | C12H9Cl | 16 |
| PCB | 2,2’-Dichlorobiphenyl (PCB 4) | 13029-08-8 | C12H8Cl2 | 16 |
| PCB | 2,2’,6-Trichlorobiphenyl (PCB19) | 38444-73-4 | C12H7Cl3 | 16 |
| PCB | 4,4’-Dichlorobiphenyl (PCB 15) | 2050-68-2 | C12H8Cl2 | 16 |
| PCB | 2,2’,6,6’-Tetrachlorobiphenyl (PCB54 | 15968-05-5 | C12H6Cl4 | 16 |
| PCB | 2,2’,4,6,6’-Pentachlorobiphenyl (PCB 104) | 56558-16-8 | C12H5Cl5 | 16 |
| PCB | 3,4,4’-Trichlorobiphenyl (PCB37) | 38444-90-5 | C12H7Cl3 | 16 |
| PCB | 2,2’,4,4’,6,6’-Hexachlorobiphenyl (PCB 155 | 33979-03-2 | C12H4Cl6 | 16 |
| PCB | 3,3’,4,4’-Tetrachlorobiphenyl (PCB 77 | 32598-13-3 | C12H8Cl4 | 16 |
| PCB | 3,4,4’,5-Tetrachlorobiphenyl (PCB 81) | 70362-50-4 | C12H6Cl4 | 16 |
| PCB | 2’,3,4,4’,5-Pentachlorobiphenyl (PCB 123) | 65510-44-3 | C12H5Cl5 | 16 |
| PCB | 2,3’,4,4’,5-Pentachlorobiphenyl (PCB 118) | 31508-00-6 | C12H5Cl5 | 16 |
| PCB | 2,3,4,4’,5-Pentachlorobiphenyl (PCB 114) | 74472-37-0 | C12H5Cl5 | 16 |
| PCB | 2,2’,3,4’,5,6,6’-Heptachlorobiphenyl (PCB 188 | 74487-85-7 | C12H3Cl7 | 16 |
| PCB | 2,2’,3,4,4’,5’ -Hexachlorobiphenyl (PCB 138) | 52712-04-6 | C12H4Cl6 | 16 |
| PCB | 2,3,3’,4,4’-Pentachlorobiphenyl (PCB 105) | 32598-14-4 | C12H5Cl5 | 16 |
| PCB | 3,3’,4,4’,5-Pentachlorobiphenyl (PCB 126) | 57465-28-8 | C12H5Cl5 | 16 |
| PCB | 2,3’,4,4’,5,5’-Hexachlorobiphenyl (PCB 167) | 52663-72-6 | C12H4Cl6 | 16 |
| PCB | 2,2’,3,3’,5,5’,6,6’-Octachlorobiphenyl (PCB 202) | 2136-99-4 | C12H2Cl8 | 16 |
| PCB | 2,3,3’,4,4’,5-Hexachlorobiphenyl (PCB 156) | 38380-08-4 | C12H4Cl6 | 16 |
| PCB | 2,3,3’,4,4’,5’-Hexachlorobiphenyl (PCB 157) | 69782-90-7 | C12H4Cl6 | 16 |
| PCB | 3,3’,4,4’,5,5’-Hexachlorobiphenyl (PCB 169) | 32774-16-6 | C12H4Cl6 | 16 |
| PCB | 2,3,3’,4,4’,5,5’-Heptachlorobiphenyl (PCB 189) | 39635-31-9 | C12H3Cl7 | 16 |
| PCB | 2,2’,3,3’,4,5,5’,6,6’-Nonachlorobiphenyl (PCB 208 | 52663-77-1 | C12HCl9 | 16 |
| PCB | 2,3,3’,4,4’,5,5’,6-Octachlorobiphenyl (PCB 205 | 74472-53-0 | C12H2Cl8 | 16 |
| PCB | 2,2’,3,3’,4,4’,5,5’,6-Nonachlorobiphenyl (PCB 206) | 40186-72-9 | C12HCl9 | 16 |
| PCB | 2,2’,3,3’,4,4’,5,5’,6,6’-Decachlorobiphenyl (PCB 209) | 2051-24-3 | C12Cl10 | 16 |
| PcDD | 2,3,7,8-Tetrachlorodibenzo-p-dioxin (TCDD) | 1746-01-6 | C12H4Cl4O2 | 6.7 |
| PcDD | 1,2,3,7,8-Pentachlorodibenzo-p-dioxin (PnCDD/PeCDD) | 40321-76-4 | C12H3Cl5O2 | 16.8 |
| PcDD | 1,2,3,6,7,8-Hexachlorodibenzo-p-dioxin (HxCDD) | 57653-85-7 | C12H2Cl6O2 | 16.8 |
| PcDD | 1,2,3,4,6,7,8-Heptachlorodibenzo-p-dioxin (HpCDD) | 35822-46-9 | C12HCl7O2 | 16.8 |
| PcDD | 1,2,3,4,6,7,8,9-Octachlorodibenzo-p-dioxin (OCDD) | 3268-87-9 | C12Cl8O2 | 33.6 |
| PcDF | 2,3,7,8-Tetrachlorodibenzofuran (TCDF) | 51207-31-9 | C12H4Cl4O | 6.7 |
| PcDF | 1,2,3,7,8-Pentachlorodibenzofuran (PnCDF/PeCDF) | 57117-41-6 | C12H3Cl5O | 16.8 |
| PcDF | 1,2,3,6,7,8-Hexachlorodibenzofuran (HxCDF) | 57117-44-9 | C12H2Cl6O | 16.8 |
| PcDF | 1,2,3,4,6,7,8-Heptachlorodibenzofuran (HpCDF) | 67562-39-4 | C12HCl7O | 16.8 |
| PcDF | 1,2,3,4,6,7,8,9-Octachlorodibenzofuran (OCDF) | 39001-02-0 | C12Cl8O | 33.6 |
| Phthalate | Dimethyl phthalate | 131-11-3 | C10H10O4 | 28 |
| Phthalate | Diethyl phthalate | 84-66-2 | C12H14O4 | 28 |
| Phthalate | Diisobutyl phthalate | 84-69-5 | C16H22O4 | 28 |
| Phthalate | Di-n-butyl phthalate | 84-74-2 | C16H22O4 | 28 |
| Phthalate | Diisohexyl phthalate | 84-63-9 | C20H30O4 | 28 |
| Phthalate | Diethoxyethyl phthalate | 605-54-9 | C16H22O6 | 28 |
| Phthalate | Dipentyl phthalate | 131-18-0 | C18H26O4 | 28 |
| Phthalate | Dihexyl phthalate | 84-75-3 | C20H30O4 | 28 |
| Phthalate | Benzyl butyl phthalate | 85-68-7 | C19H20O4 | 28 |
| Phthalate | Di(2-butoxyethyl) phthalate | 117-83-9 | C20H30O6 | 28 |
| Phthalate | Dicyclohexyl phthalate | 84-61-7 | C20H26O4 | 28 |
| Phthalate | Di-2-ethylhexyl phthalate | 117-81-7 | C24H38O4 | 28 |
| Phthalate | Diphenyl phthalate | 84-62-8 | C20H14O4 | 28 |
| Phthalate | Di-n-octyl phthalate | 117-84-0 | C24H38O4 | 28 |
| Phthalate | Dinonyl phthalate | 84-76-4 | C26H42O4 | 28 |
| Labeled PAH | Acenaphthene-d10 | -- | C12D10 | 28 |
| Labeled PAH | Fluorene-d10 | -- | C13D10 | 28 |
| Labeled PAH | Phenanthrene-d10 | -- | C14D10 | 28 |
| Labeled PAH | Anthracene-d10 | -- | C14D10 | 28 |
| Labeled PAH | Fluoranthene-d10 | -- | C16D10 | 28 |
| Labeled PAH | Pyrene-d10 | -- | C16D10 | 28 |
| Labeled PAH | Benz[a]anthracene-d12 | -- | C18D12 | 28 |
| Labeled PAH | Chrysene-d12 | -- | C18D12 | 28 |
| Labeled PAH | Benzo[b]fluoranthene-d12 | -- | C20D12 | 28 |
| Labeled PAH | Benzo[k]fluoranthene-d12 | -- | C20D12 | 28 |
| Labeled PAH | Benzo[a]pyrene-d12 | -- | C20D12 | 28 |
| Labeled PAH | Indeno[1,2,3-cd]pyrene-d12 | -- | C22D12 | 28 |
| Labeled PAH | Dibenzo[a,h]anthracene-d14 | -- | C22D14 | 28 |
| Labeled PAH | Benzo[g,h,i]perylene-d12 | -- | C22D12 | 28 |
| Labeled PAH | Naphthalene-d8 | -- | C10D8 | 28 |
| Labeled PAH | Acenaphthylene-d8 | -- | C12D8 | 28 |
| Labeled PCB | 2,4,4’-Trichlorobiphenyl-13C12 (PCB 28) | -- | C12H7Cl3 | 10 |
| Labeled PCB | 2,2’,5,5’-Tetrachlorobiphenyl-13C12 (PCB 52) | -- | 13C12H6Cl4 | 10 |
| Labeled PCB | 2,2’,4,5,5’-Pentachlorobiphenyl-13C12 (PCB 101) | -- | 13C12H5Cl5 | 10 |
| Labeled PCB | 2,2’,3,4,4’,5’ -Hexachlorobiphenyl-13C12 (PCB 138) | -- | 13C12H4Cl6 | 10 |
| Labeled PCB | 2,2’,4,4’,5,5’-Hexachlorobiphenyl-13C12 (PCB 153) | -- | 13C12H4Cl6 | 10 |
| Labeled PCB | 2,2’,3,4,4’,5,5’-Heptachlorobiphenyl-13C12 (PCB 180) | -- | 13C12H3Cl7 | 10 |

**Table S1B. List of native and isotopic labeled standards used for targeted analysis (Level 1 identifications) in air samples.** Acronyms: Brominated flame retardants (BFR); hydrocarbon (HC); organochlorine pesticides (OCP); organophosphates (OPE); polycyclic aromatic hydrocarbons (PAH); polychlorinated biphenyls (PCB); VOC: volatile organic compounds (VOC).

| **Chemical Class** | **Detected Metabolite** | **CAS-RN** | **Molecular Formula** |
| --- | --- | --- | --- |
| Alkaloid | Nicotine | 54-11-5 | C10H14N2 |
| Benzodioxole | Fludioxonil | 131341-86-1 | C12H6F2N2O2 |
| Benzopyran | Delta-9-tetrahydrocannabinol | 1972-08-3 | C21H30O2 |
| BFR | 2,4,4'-Tribromodiphenyl ether (BDE-28) | 41318-75-6 | C12H7Br3O |
| BFR | 2,2',4,4'-Tetrabromodiphenyl ether (BDE-66) | 5436-43-1 | C12H6Br4O |
| BFR | 2,2',4,4',5-Pentabromodiphenyl ether (BDE-99) | 60348-60-9 | C12H5Br5O |
| BFR | 2,2',4,4',6-Pentabromodiphenyl ether (BDE-100) | 189084-64-8 | C12H5Br5O |
| BFR | 2,2',4,4',5,5'-Hexabromodiphenyl ether (BDE-153) | 68631-49-2 | C12H4Br6O |
| BFR | 2,2',4,4',5,6'-Hexabromodiphenyl ether (BDE-154) | 207122-15-4 | C12H4Br6O |
| BFR | 2-Ethylhexyl 2,3,4,5-tetrabromobenzoate | 183658-27-7 | C15H18Br4O2 |
| Chlorinated HC | 1,2,4-Trichlorobenzene | 120-82-1 | C6H3Cl3 |
| Chlorinated HC | Hexachlorobutadiene (HCBD) | 87-68-3 | C4Cl6 |
| Chlorinated HC | Hexachlorocyclopentadiene | 77-47-4 | C5Cl6 |
| Chlorinated HC | Hexachloroethane | 67-72-1 | C2Cl6 |
| Haloethers | Bis(2-chloro-1-methylethyl) ether (BCEE) | 108-60-1 | C6H12Cl2O |
| Haloethers | Bis(2-chloroethyl)ether | 111-44-4 | C4H8Cl2O |
| Haloethers | Carfentrazone-ethyl | 128639-02-1 | C15H14Cl2F3N3O3 |
| Nitroaromatic | 2,4-Dinitrotoluene | 121-14-2 | C7H6N2O4 |
| Nitroaromatic | 2,6-Dinitrotoluene | 606-20-2 | C7H6N2O4 |
| Nitroaromatic | 4-Chloroaniline | 106-47-8 | C6H6ClN |
| Nitroaromatic | 4-Nitroaniline | 100-01-6 | C6H6N2O2 |
| Nitroaromatic | Isophorone | 78-59-1 | C9H14O |
| Nitrosoamine | Nitrobenzene | 98-95-3 | C6H5NO2 |
| Nitrosoamine | N-Nitrosodi-n-propylamine | 621-64-7 | C6H14N2O |
| Nitrosoamine | N-Nitrosodiphenylamine | 86-30-6 | C12H10N2O |
| OCP | alpha-Hexachlorocyclohexane (α-HCH) | 319-84-6 | C6H6Cl6 |
| OCP | Chlorothalonil | 1897-45-6 | C8Cl4N2 |
| OCP | Dieldrin | 60-57-1 | C12H8Cl6O |
| OCP | Endosulfan I | 959-98-8 | C9H6Cl6O3S |
| OCP | Endrin | 72-20-8 | C12H8Cl6O |
| OCP | gamma-Hexachlorocyclohexane (γ-HCH) | 58-89-9 | C6H6Cl6 |
| OCP | Hexachlorobenzene | 118-74-1 | C6Cl6 |
| OCP | Methoxychlor | 72-43-5 | C16H15Cl3O2 |
| OCP | p,p'-DDD | 72-54-8 | C14H10Cl4 |
| OCP | p,p'-DDT | 50-29-3 | C14H9Cl5 |
| OCP | Tetrachloro-m-xylene | 877-09-8 | C8H6Cl4 |
| OPE | Triphenyl phosphate (TPHP) | 115-86-6 | C18H15O4P |
| OPE | Tris(1-chloro-2-propyl) phosphate (TCPP) | 13674-84-5 | C9H18Cl3O4P |
| PAH | 1-Bromo-4-phenoxybenzene | 101-55-3 | C12H9BrO |
| PAH | 1-Chloro-4-phenoxybenzene | 7005-72-3 | C12H9ClO |
| PAH | 2-Chloronaphthalene | 91-58-7 | C10H7Cl |
| PAH | 2-Methylnaphthalene | 91-57-6 | C11H10 |
| PAH | Acenaphthene | 83-32-9 | C12H10 |
| PAH | Acenaphthylene | 208-96-8 | C12H8 |
| PAH | Anthracene | 120-12-7 | C14H10 |
| PAH | Benz[a]anthracene | 56-55-3 | C18H12 |
| PAH | Benzo[a]pyrene | 50-32-8 | C20H12 |
| PAH | Benzo[b]fluoranthene | 205-99-2 | C20H12 |
| PAH | Benzo[ghi]perylene | 191-24-2 | C22H12 |
| PAH | Benzo[k]fluoranthene | 207-08-9 | C20H12 |
| PAH | Chrysene | 218-01-9 | C18H12 |
| PAH | Dibenz[a,h]anthracene | 53-70-3 | C22H14 |
| PAH | Dibenzofuran | 132-64-9 | C12H8O |
| PAH | Fluoranthene | 206-44-0 | C16H10 |
| PAH | Fluorene | 86-73-7 | C13H10 |
| PAH | Indeno[1,2,3-cd]pyrene | 193-39-5 | C22H12 |
| PAH | Naphthalene | 91-20-3 | C10H8 |
| PAH | Phenanthrene | 85-01-8 | C14H10 |
| PAH | Pyrene | 129-00-0 | C16H10 |
| PCB | 2,2',3,3',4,4',5,5',6,6'-Decachlorobiphenyl (PCB 209) | 2051-24-3 | C12Cl10 |
| Phthalate | Bis(2-ethylhexyl) phthalate | 117-81-7 | C24H38O4 |
| Phthalate | Butylbenzyl phthalate | 85-68-7 | C19H20O4 |
| Phthalate | Diethyl phthalate | 84-66-2 | C12H14O4 |
| Phthalate | Dimethyl phthalate | 131-11-3 | C10H10O4 |
| Phthalate | Di-n-butyl phthalate | 84-74-2 | C16H22O4 |
| Phthalate | Di-n-octyl phthalate | 117-84-0 | C24H38O4 |
| Pyrethroid | Piperonyl butoxide | 51-03-6 | C19H30O5 |
| VOC | 1,2-dichlorobenzene | 95-50-1 | C6H4Cl2 |
| VOC | 1,3-dichlorobenzene | 541-73-1 | C6H4Cl2 |
| VOC | 1,4-dichlorobenzene | 106-46-7 | C6H4Cl2 |

**Figure S1**: **Molecular ions can be used to reduce the false positive rate (albeit increasing the false negative rate) as shown using a GC-HRMS dataset of air samples.**
